# A Digital Feature Recognition Technology Used in Ballet Training Action Correction

**DOI:** 10.1155/2022/7953172

**Published:** 2022-02-25

**Authors:** Jia Sun

**Affiliations:** Guangxi University of Arts, Nanning 530000, China

## Abstract

In order to improve the effect of ballet training, this paper combines digital technology to improve the motion recognition process, analyzes the imaging process, analyzes the system process combined with the human node model, and combines digital feature recognition technology to construct a ballet training motion correction system. Moreover, this paper inputs the ballet training action recognition results into the system and compares the standard actions to judge the rationality of the actions and builds a ballet training action correction system based on digital feature recognition. In addition, this paper designs experiments to conduct ballet training movements in the system effect evaluation. The experimental research verifies that the digital feature recognition technology proposed in this paper can play an important role in ballet movement recognition and has a good action correction effect.

## 1. Introduction

Ballet is a charming and refreshing art. Its elegance and beauty can be regarded as the essence of human civilization and progress, and it is praised as “the crown jewel of dance art” [1]. Its graceful dance, light jumping, and continuous toe rotation often bring people into a dreamlike artistic realm. Ballet originated in Italy during the European Renaissance, flourished in France, and flourished in Russia, and finally moved from Russia to the world. After hundreds of years of development, ballet has formed its own complete training system. In particular, it is unique in the scientific training methods of the correct sense of the feet, posture, various parts of the body, and the consciousness of movement performance [2]. The ballet training system is to improve the performer's physical ability and performance awareness through a series of complete programmatic body movement combination training. Moreover, it enables the dancers to freely and colorfully express the performers' rich thoughts and passions in various situations, thereby creating a brand-new artistic image [3].

The romantic ballet showed the body aesthetics so vividly that the criterion for judging this beauty has continued to this day. The basic skill training of Lei can be summarized as the four principles of opening, stretching, upright, and standing, which are basically consistent with the requirements of the competition rules for the body technical movement specifications of the artistic performances. Basic ballet training has a positive effect on the aesthetics and standardization of Latin dance. Therefore, the basic training of ballet is very important for the study and training of Latin dance. At present, there are still many problems that need to be improved in actual teaching: firstly, there is no ballet training material suitable for special and unified sports dance; secondly, the teaching hours are generally too short to meet the special requirements; finally, the goal of ballet training is pertinent not strong, ignoring the training needs of each item. Only by further studying the value and role of basic ballet training in Latin dance teaching, strengthening the thinking of the degree of integration of basic dance and professional dance, and improving the awareness of basic dance services for special dances, can the teaching effect be better improved.

Ballet dynamic technology is to improve the dancers' technical movements and physical abilities through a series of complete and standardized body movements, so as to better show the dance style and dance performance ability, so that the dancers can be in a variety of music rhythms. This kind of emotions can show graceful dance with ease, convey the most accurate emotional expression to the audience, show the most exquisite technical movements, and have the characteristics of science and standardization. Ballet dynamic techniques cover jumping, squatting, turning, and other techniques. In foreign countries, many modern dancers have been trained in ballet since they were young, so they have solid ballet dynamic skills, making the basic dance skills much better than ordinary people, and the performance of modern dance competitions is also very good.

This article combines digital feature recognition technology to carry out ballet training movement correction analysis and combines the actual ballet movement to correct the actual needs for research and proposes a corresponding intelligent training correction system to improve the effect of ballet training.

## 2. Related Work

Literature [4] introduced the importance of ballet in the dance classroom and then analyzed the problems of unreasonable curriculum setting, single teaching method, and insufficient teachers in the reform of ballet classroom teaching. In response to these problems, corresponding solutions are put forward to help the ballet classroom to be better developed and play its role. Literature [5] pointed out that ballet is a must-have course for dance practitioners. It can not only affect and improve the beauty of people, but also make people have beauty of posture and temperament. It is precisely because of a scientific and standardized system such as ballet that the flowers of dance art can bloom more brilliantly. Literature [6] mentioned that the technical level of modern ballet can be divided into dynamic technology and static technology. Similarly, sports dance also includes dynamic movements and static movements. Dynamic movements require agility and coordination, which not only involves complex steps, but also requires physical sensations and delicate and changeable techniques. Literature [7] believed that the basic ballet training course is an indispensable course for dance majors, and it is also a major course in the ballet teaching system. The most prominent feature is that it not only trains various positions of the body, but also plays a comprehensive training role in the coordination of the entire body. Gao Yiyan said that dance is the art of the body, and the courses to exercise the dancers' bodies are very important. The reason why basic ballet training can be regarded as one of the compulsory basic courses for all dance majors is that basic ballet training is the longest and most scientific basic training course in history. The purpose of the basic ballet training course is to train students' muscles, skills, abilities, etc., to help students lay a foundation for future performances [8].

Literature [9] pointed out that there are several problems in the development process of dance movement in the emerging stage, such as the decline of dance movement athletes' physical fitness, low technical level, unnew arrangement, and poor completion, and proposed development strategies to improve dance movement items. First of all, establish a scientific and complete training plan, and insist on scientific training for a long time. Secondly, strengthen the coach's own quality and coaching ability, continue to promote and popularize dance movement training, and expand the number of reserve talents to provide talents for the national team. Literature [10] made a preliminary popularization of the origin and development form of dance movements to other scholars who did not understand the development of dance movements and let people gradually understand the development history and essential characteristics of dance movements. The article mentioned that both now and in the future, only the combination of sports and art can continuously create new results. In particular, modern people pursue a healthy lifestyle and the beauty of sports. Therefore, it is necessary to strengthen the popularization of people to shape a healthy lifestyle through sports. Literature [11] proposes that only coaches should follow the latest trends in the development of dance movement items and, on the premise of accurately understanding and grasping the scoring rules, propose and implement corresponding training for the common problems of dance movement athletes at this stage and the athletes' physical conditions. Only plans and countermeasures can adapt to the rules of international dance movement competitions and promote the development of dance movement projects in the direction of art and competition that is difficult and beautiful. Literature [12] refers to the limitation of sports events on the road to the emergence of institutional development, forcing sports events to be unable to continue their development.

Literature [13] writes that dance moves are still a young athletic event, so there is a lot of room for improvement. The complete set of dance moves is not only a superimposition of difficulty, but also a perfect match with the equipment. Therefore, the individual characteristics of the project should be fully considered during training, combined with the athlete's physical ability, and appropriate auxiliary method training should be strengthened, including basic quality training such as flexibility, strength, speed, endurance, agility, etc. It also includes the characteristics of different equipment for training to integrate and complement each other. Literature [14] writes that coaches emphasize physical fitness while neglecting equipment skills in training. Poor proficiency directly affects the complete quality of the entire set. If the proficiency training in equipment movements, continuity, and coordinated movements is not strengthened, it will be restricted. For the progress of the dance movement project, [15] pointed out that it has begun to pay attention to the research of athlete selection, movement arrangement, physical training, etc. and strictly train outstanding athletes. Literature [16] writes that it is important to scientifically and rationally arrange the training plans for each stage of the small, medium, and large cycles, intersperse competitions, assessments, relaxation, and other links in the quality training, and combine the large and small cycles, so that the cycle training can achieve excellent results. Literature [17] emphasizes the balanced development of various difficult movements, attaches importance to difficult movements and balance movements, strictly regulates movement standards, strengthens the time of equipment training, participates in more international competitions, accumulates experience, and makes use of the good model of competition-driven training.

## 3. Digital Feature Recognition Technology

Multiview dense matching uses homography to perform dual-view stereo matching between multiple cameras. The homography relationship can be expressed as follows: a point in a certain image plane can determine the position of its corresponding point in the second image plane through the homography induced by a certain plane.

**Figure 1:**
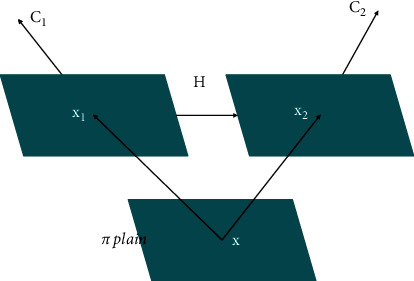
The homography induced by plane in dual-viewpoint matching.

As shown in Figure 1, a point *x*_1_ in the image plane of the camera *C*_1_ can be used to determine the position of the matching point *x*_2_ in the image plane of the camera *C*_2_ through the homography *H* induced by the *π* plane. The calculation method of the homography matrix *H* is as follows:

We assume that the projection matrices of camera *C*_1_ and camera *C*_2_ are *P*_1_ and *P*_2_, respectively. First, we back-project the plane point *x*, and the back-projection ray intersects the plane *π* at the three-dimensional point *X*, so there is *x*_1_=*P*_1_*X*. Then, we map the three-dimensional point *X* projection to the plane point *x*_2_ and denote it as *x*_2_=*P*_2_*X*. At this time, the homography correspondence between the plane points *x*_1_ and *x*_2_ can be obtained as [18]

(1) $$\begin{array}{c} x_{2}=P_{2}X=P_{2}P_{1}^{-1}x_{1}=Hx_{1}. \end{array}$$

We assume that the projection matrices of camera *C*_1_ and camera *C*_2_ are *P*_1_=*K*_1_[*I|*0] and *P*_2_=*K*_2_[*R|t*], respectively, and the *π* plane can be expressed as *π*=(*n*^*T*^, 1)^*T*^ (*n* is a certain unit vector). Any three-dimensional point on the projection ray of the plane point *x*1 can be expressed as *x*=(*x*_1_, *η*)^*T*^, where *η* is the parameterized coefficient of the point *X* on the projection ray. Since the three-dimensional point *X* is on the plane *π* at the same time, there is *π*^*T*^*X*=0, which is transformed into *X*=(*x*_1_, −*n*^*T*^*x*_1_)^*T*^. Therefore, formula (1) can be further expressed as [19]

(2) $$\begin{array}{c} x_{2}=P_{2}X=P_{2}{\left(x_{1},-n^{T}x_{1}\right)}^{T}\\ =K_{2}\left(R-tn^{T}\right)K_{1}^{-1}x_{1},\\ K=K_{2}\left(R-tn^{T}\right)K_{1}^{-1}. \end{array}$$

It is composed of the internal parameter matrix of the camera, the relative external parameters between the cameras, and the plane parameters.

**Figure 2:**
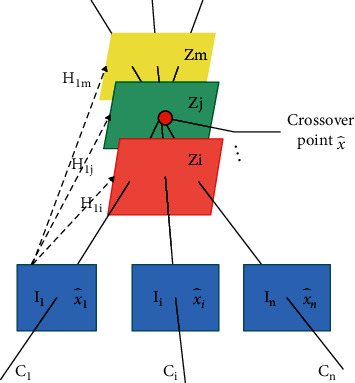
Schematic diagram of the principle of Collins planar scanning algorithm.

The schematic diagram of the spatial plane scanning algorithm is shown in Figure 2.

In Figure 2, we assume that there are a multicamera image *I*_*i*_ (*i*=1,…, *n*) and a series of parallel virtual planes *Z*_*j*_ (*j*=1,…, *m*) scanned along the *Z* axis in space. In the algorithm, each virtual plane is divided into cells of a certain size, and the cell size determines the accuracy of the three-dimensional points in the reconstruction result. The principle of the spatial plane scanning algorithm is that the algorithm back-projects the image feature point ${\hat{x}}_{i}$, and the back-projected rays intersect the corresponding cell of each virtual plane. The algorithm performs back-projection operation on all image feature points and records the number of times that all cells are intersected. The algorithm counts the total number of intersections of each cell, and the cell with the number of intersections greater than the preset threshold is regarded as the three-dimensional point $\hat{X}$ corresponding to the multiview matching. At the same time, the image point ${\hat{x}}_{i}\;\left(i=1,\ldots ,n\right)$ corresponding to the three-dimensional point transmission projection is the matching point of the multiview image.

The image point ${\hat{x}}_{i}{\left(x,y\right)}^{T}$ and the three-dimensional point $\hat{X}={\left(X,Y,Z,1\right)}^{T}$ in space are both representations of homogeneous coordinates. The process of projecting the point $\hat{X}$ in space onto a certain camera image plane *I*_*i*_ is [20]

(3) $$\begin{array}{c} {\hat{x}}_{i}=K\left[\begin{array}{cccc} r_{1} & r_{2} & r_{3} & t \end{array}\right]\hat{X}. \end{array}$$

*K* in the equation is the camera's internal parameter matrix.

$R=\left[\begin{array}{ccc} r_{1} & r_{2} & r_{3} \end{array}\right]$ and *t* are the external parameters of the camera. Since the three-dimensional point $\hat{X}$ in space lies on the plane *Z*_*f*_, (3) can be replaced by

(4) $$\begin{array}{c} \left[\begin{array}{c} x\\ y\\ 1 \end{array}\right]=K\left[\begin{array}{ccc} r_{1} & r_{2} & Z_{j}r_{3}+t \end{array}\right]\left[\begin{array}{c} X\\ Y\\ 1 \end{array}\right]. \end{array}$$

In this way, the projective transformation is transformed into a homography transformation of two planes, and the homography matrix *H*_*j*_ is [21]

(5) $$\begin{array}{c} H_{j}=K\left[\begin{array}{ccc} r_{1} & r_{2} & Z_{j}r_{3}+t \end{array}\right]. \end{array}$$

In order to calculate the intersection position of the back-projection ray of the image point and all virtual planes more effectively, we propose to precalculate the intersection (*X*_1_, *Y*_1_, *Z*_1_)^*T*^ of the back-projection ray of the image point and a certain virtual plane *Z*_1_. After that, we directly perform a homography transformation on (*X*_1_, *Y*_1_, *Z*_1_)^*T*^ to solve the intersection point (*X*_*j*_, *Y*_*j*_, *Z*_*j*_) between the back-projected ray and other virtual planes, instead of retransforming the source image point. The calculation expression is

(6) $$\begin{array}{c} \left(X_{j},Y_{j},Z_{j}\right)=H_{j}^{-1}H_{1}\left(X_{1},Y_{1},Z_{1}\right). \end{array}$$

We assume a pixel *p* and its surrounding neighborhood pixel square *N*(*p*); the intensity of the pixel is expressed as *I*(*p*). Nonparametric local transformation is to compare the intensity value *I*(*p*′) of pixel *p*′(*p*′ ∈ *N*(*p*)) in the neighborhood square matrix with *I*(*p*) and define the sign as

(7) $$\begin{array}{c} \xi \left(p,p{\;}^{\prime }\right)=\left\{\begin{array}{cc} 1, & I\left(p{\;}^{\prime }\right)<I\left(p\right),\\ 0, & I\left(p{\;}^{\prime }\right)>I\left(p\right). \end{array}\right) \end{array}$$

On this basis, the Rank transformation is expressed as

(8) $$\begin{array}{c} R\left(p\right)=\left\|\left\{p{\;}^{\prime }\in N\left(p\right)|\xi \left(p,p{\;}^{\prime }\right)=1\right\}\right\|. \end{array}$$

It refers to the number of pixels in the neighborhood square matrix whose pixel intensity value is less than the center pixel intensity value. When calculating region matching, the *L*I norm relationship is used for minimization, and the center pixel corresponding to the region with the smallest number after comparison is selected as the matching pixel. It directly performs hierarchical similarity comparison of color images and adopts a multiwindow comparison method to improve the matching accuracy in the case of discontinuous parallax.

Different from the Rank transform, the idea of the Census transform is to compare the intensity value difference between the pixels in the neighborhood square matrix and the center pixel and express the comparison result as a binary bit string in order. The mathematical expression of this transformation is

(9) $$\begin{array}{c} C\left(p\right)=\underset{p{\;}^{\prime }\in N\left(P\right)}{⊗}\xi \left(p{\;}^{\prime },p\right). \end{array}$$

**Figure 3:**
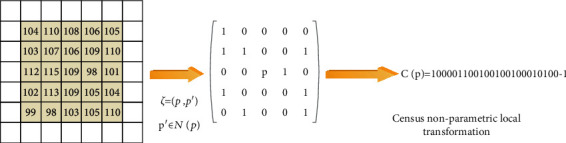
Census nonparametric local transformation.

In the formula, the symbol ⊗ represents the concatenation of each value *ξ*(*p*′, *p*). The schematic diagram of the Census transformation is shown in Figure 3, where the pixels in the red dashed frame in the figure are the neighborhood pixel square matrix *N*(*p*). Because the Census transform not only uses nonparametric binary characters to represent the attributes of the pixels, but also incorporates the order of the neighboring pixel attributes into the constraints, it is more robust than the Rank transform in the face of changes in external lighting conditions.

After the Census transformation of the multiview image to be matched, the Hamming distance between the pixels to be matched is calculated to achieve multiview stereo matching. The Hamming distance refers to the number of different bits between two bit strings. It is very intuitive to express the degree of similarity between pixels. The method of using Hamming distance as the matching criterion can be expressed as

(10) $$\begin{array}{c} \text{Cost}\left(p,d\right)=\text{Hamming}\left(C_{1}\left(p\right),C_{2}\left(p+d\right)\right). \end{array}$$

In the formula, Cost(*p d*) represents the matching cost of the pixel *p* to be matched at the disparity *d*, and *C*_1_ and *C*_2_ are the bit strings of the two viewpoint images after the Census transformation.

In the case of discontinuous parallax, usually a point-by-point matching combined with global optimization is used for processing to reduce the erroneous matching caused by noise and obtain more accurate matching results. The global optimization in this method adds a smoothing term as an additional constraint condition on the basis of the matching cost, so as to punish the change of the neighborhood parallax, which can be expressed mathematically as

(11) $$\begin{array}{c} E\left(D\right)=\sum_{p}\left(C\left(p,D_{p}\right)\right)+\sum_{p{\;}^{\prime }\in N_{p}}pT\left[\left|D_{p}-D_{p^{\prime }}\right|\geq 1\right]. \end{array}$$

When the neighborhood disparity change |*D*_*p*_ − *D*_*p*′_| is greater than or equal to one pixel, a constant penalty coefficient *p* is added to preserve the discontinuity of the disparity. The stereo matching problem combined with the global method becomes the problem of minimizing the global energy function *E*(*D*) and solving the parallax *D*. However, (11) is a two-dimensional global energy function, and it is a complicated process to minimize it. Although the idea of dynamic programming can be used to efficiently minimize one-dimensional image rows one by one, it is still difficult to achieve optimal association between rows.

Different from the energy function of global matching, the energy function of semiglobal matching uses two smoothing coefficients *p*1 and *p*2 of different sizes to penalize different degrees of parallax changes, which is mathematically expressed as

(12) $$\begin{array}{c} E\left(D\right)=\sum_{p}\left(C\left(p,D_{p}\right)\right)+\sum_{p^{\prime }\in N_{p}}pT\left[\left|D_{p}-D_{p^{\prime }}\right|=1\right]+\sum_{p^{\prime }\in N_{p}}pT\left[\left|D_{p}-D_{p^{\prime }}\right|>1\right]. \end{array}$$

The *p*1 in formula (12) penalizes a small range of parallax (for example, the parallax is one pixel) and can adaptively preserve the inclined surface or curved surface, while *p*2, which penalizes large parallax, can preserve the discontinuous area. The most important thing is that the semiglobal matching adopts a new matching cost accumulation method, which equally accumulates the one-dimensional matching cost of all directions of the pixels, and uses the minimum cost sum of all directions as the final cumulative matching cost. The minimum cost of a path in a certain direction can be expressed as

(13) $$\begin{array}{c} {\text{Cost}}_{r}\left(p,d\right)=C\left(p,d\right)+\min\left(Cost_{r}\left(p-r,d\right),{\text{Cost}}_{r}\left(p-r,d-1\right)+P_{1}\right)\left({\text{Cost}}_{r}\left(p-r,d+1\right)+p_{1}\underset{r}{\min}{\text{Cost}}_{r}\left(p-r,i\right)+p_{2}\right). \end{array}$$

In the formula, *C*(*p*,*d*) represents the point-by-point matching cost, *r* represents the directional path, *p*−*r* is the pixel before the pixel in the *r* direction, and *d* represents the parallax. The final cumulative matching cost is expressed as

(14) $$\begin{array}{c} {\text{Cost}}_{\text{agg}}\left(p,d\right)=\sum_{r}{\text{Cost}}_{r}\left(p,d\right). \end{array}$$

**Figure 4:**
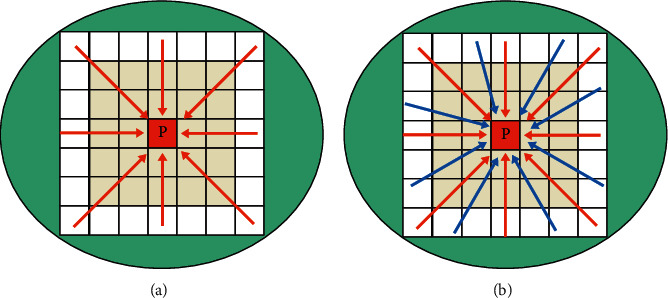
The cumulative path of semiglobal matching costs: (a) 8-direction path; (b) 16-direction path.

The actual application process usually accumulates the minimum cost of 8 or 16 directional paths, as shown in Figure 4. The paths in the eight directions are row accumulation, column accumulation, and diagonal accumulation. In addition to the rows, columns, and diagonals of the 16-direction paths, the accumulation method of the remaining 8 directions is to first perform row or column accumulation, then perform diagonal accumulation, and perform alternately. Finally, the cumulative matching cost energy function Cost_agg_(*p*, *d*) is minimized, and the matching disparity value *d* corresponding to each pixel is obtained, and the matching is completed.

In the field of computer vision, applications such as image denoising, stereo image dense matching, and depth generation all have erroneous estimates due to external noise. For this reason, outliers are usually eliminated. The principle of outlier removal can be understood from the perspective of probability models:

$\hat{g}\left(x\right)$ is a series of noisy measurement values; *u*(*x*) ∈ *R*^*M*×*N*^ is the solution of the required model. According to Bayesian inference, the probability of obtaining the model solution *u*(*x*) optimized from the measurement value *b* is expressed as

(15) $$\begin{array}{c} p\left(u|\hat{g}\right)=\frac{p\left(\hat{g}|u\right)p\left(u\right)}{p\left(\hat{g}\right)}. \end{array}$$

In formula (15), $p\left(\hat{g}|u\right)$ is called the data generation model, and *p*(*u*) is called the prior model. The denominator $p\left(\hat{g}\right)$ is a constant when the measured value is known, so $p\left(u|\hat{g}\right)$ is proportional to $p\left(\hat{g}|u\right)p\left(u\right)$ and can be expressed as $p\left(\hat{g}|u\right)\propto p\left(\hat{g}|u\right)p\left(u\right)$. Therefore, the target solution $\tilde{u}$ can be solved by maximizing the posterior probability $p\left(u|\hat{g}\right)$:

(16) $$\begin{array}{c} \tilde{u}=\underset{u}{\max}p\left(\hat{g}|u\right)p\left(u\right). \end{array}$$

Most practical engineering applications will use global optimization methods to eliminate outliers. The global optimization method sets $E\left(u\right)=-\ln\;p\left(u|\hat{g}\right)$ to convert the probability maximization problem to the energy minimization problem. There are

(17) $$\begin{array}{c} E\left(u\right)=-\ln\;p\left(u|\hat{g}\right)\propto -\ln\;p\left(\hat{g}|u\right)\cdot -\ln\;p\left(u\right),\\ \tilde{u}=\underset{u}{\min}\left\{E\left(u\right)\right\}. \end{array}$$

Furthermore, global optimization usually replaces the probability model with a general energy model, which consists of data items containing model solutions and regular items with smoothing effect, expressed as

(18) $$\begin{array}{c} E\left(u\right)≜D\left(u|\hat{g}\right)+\tau R\left(u\right),\\ \tilde{u}=\underset{u}{\min}\left\{E\left(u\right)\right\}. \end{array}$$

The data item $E\left(u\right)=\int_{\Omega }\psi_{D}\left(es\left(u|x\right),\hat{g}\left(x\right)\right)\text{d}x+\tau \int_{\Omega }\psi_{D}\left(\nabla u\left(x\right)\right)\text{d}x$ in equation (19) is proportional to −ln  *p*(*u*), and the regular term *R*(*u*) is proportional to $D\left(u|\hat{g}\right)$. The data item consists of the error function *e* induced by the model solution *u*(*x*) and the measured value $\hat{g}\left(x\right)$ and the positive penalty function *ψ*_*D*_, expressed as

(19) $$\begin{array}{c} D\left(u|\hat{g}\right)=\sum_{x\in \Omega }\psi_{R}\left(e\left(u\left(x\right)\right),\hat{g}\left(x\right)\right). \end{array}$$

The regular term is composed of the smooth function of the model solution *u*(*x*) and the positive penalty function *v*, expressed as

(20) $$\begin{array}{c} R\left(u\right)=\sum_{x\in \Omega }\psi_{R}\left(s\left(u\left(x\right)\right)\right). \end{array}$$

The value of the regular term is small, which plays a role in preserving certain characteristics of the target solution. In the continuous domain, the smoothing of the model solution *u*(*x*) can be calculated by *s*(*u|x*)=∇*u*(*x*).

Finally, a variational global optimization energy model can be defined to solve the problem of removing outliers. The energy model is mathematically expressed as

(21) $$\begin{array}{c} E\left(u\right)=\int_{\Omega }\psi_{D}\left(es\left(u|x\right),\hat{g}\left(x\right)\right)\text{d}x+\tau \int_{\Omega }\psi_{D}\left(\nabla u\left(x\right)\right)\text{d}x. \end{array}$$

The solution method of formula (21) is to use the Euler-Lagrangian equation ∂*E*(*u*)/∂*u*=0 which minimizes the energy functional to find the minimum value of the model solution *u*(*x*). In particular, when both the data item and the regular item are convex, the model solution obtained by ∂*E*(*u*)/∂*u*=0 is the minimum value.

When a convex function is not differentiable at a certain point, the problem of nondifferentiation is often solved by solving its corresponding convex conjugate. The specific method is as follows: by the Legendre-Fenchel transformation, the convex conjugate of the original function *f*(*x*) can be written as

(22) $$\begin{array}{c} f^{*}\left(y\right)=\underset{x\in R^{u}}{\sup}\left\{\langle x,y\rangle -f\left(x\right)\right\}. \end{array}$$

Among them, *y* is a dual variable, and the symbol <…> means calculating the inner product of the original variable *x* and the dual variable *y*, and the transformed function is differentiable in the entire domain *y*. Therefore, the problem that the original *L*l norm is not differentiable at *x* = 0 can be solved by convex coballoons. The convex conjugation of *L*l cost |*x*|_1_ is

(23) $$\begin{array}{c} f^{*}\left(y\right)=\delta \left(y\right). \end{array}$$

In the formula, *δ*(*y*) is the indicator function, and when ‖*y*‖_1_ ≤ 1, *δ*(*y*)=0. At the same time, the dual form of the original *L*I norm can be written as

(24) $$\begin{array}{c} {\left|x\right|}_{1}=\underset{y\in R^{n},{\left\|y\right\|}_{\infty }<1}{\max}\left\{\langle x,y\rangle -\delta \left(y\right)\right\}. \end{array}$$

Similarly, the convex conjugate combined with the quadratic cost *x*^2^ is

(25) $$\begin{array}{c} f^{*}\left(y\right)=\frac{\varepsilon }{2}{\left\|y\right\|}_{2}^{2},\\ \forall {\left\|y\right\|}_{2}\leq \varepsilon . \end{array}$$

The dual forms of the original quadratic norm and the original Huber norm are

(26) $$\begin{array}{c} \left\{\begin{array}{c} {\left\|x\right\|}_{2}=\underset{y\in R^{n},{\left\|y\right\|}_{2}<\varepsilon }{\max}\left\{\langle x,y\rangle -\frac{\varepsilon }{2}{\left\|y\right\|}_{2}^{2}\right\}\left(a\right),\\ {\left\|x\right\|}_{2}=\underset{y\in R^{n},{\left\|y\right\|}_{2}<1}{\max}\left\{\langle x,y\rangle -\delta \left(y\right)-\frac{\varepsilon }{2}{\left\|y\right\|}_{2}^{2}\right\}\left(b\right). \end{array}\right) \end{array}$$

We assume that the expression of a common saddle point problem is

(27) $$\begin{array}{c} \underset{x\in X}{\min}\underset{y\in Y}{\max}\langle Lx,y\rangle +G\left(x\right)-F^{*}\left(y\right). \end{array}$$

In the formula, *x* and *Y* are two-dimensional real vector spaces, and *L* is a continuous linear operation on *X* ⟶ *Y*. The symbol *F*^*∗*^ represents the convex conjugation of the function *F*. The saddle point problem can be regarded as the original problem:

(28) $$\begin{array}{c} \underset{x\in X}{\min}F\left(Lx\right)+G\left(x\right), \end{array}$$

or the original dual description of the corresponding dual problem:

(29) $$\begin{array}{c} \underset{y\in Y}{\max}-\left(F^{*}\left(y\right)+G^{*}\left(-L^{*}y\right)\right). \end{array}$$

For the optimization of this kind of saddle point problem, an iterative optimization method for solving the original variable *x* and the dual variable *y* is given. First, we obtain partial derivatives of formula (27) to obtain the presolved subformulas of *x* and *y*:

(30) $$\begin{array}{c} \left\{\begin{array}{c} x={\left(I+\nabla G\right)}^{-1}\left(x-Ly\right),\\ y={\left(I+\nabla F\right)}^{-1}\left(y+Lx\right). \end{array}\right) \end{array}$$

The symbol ∇ in the formula is the gradient operator. After that, the original variable and the dual variable are updated iteratively through the gradient descent/rise method. Taking the ROF denoising model as an example, the expression of the original energy model is

(31) $$\begin{array}{c} \underset{u\in R^{MN}}{\min}E\left(u\right)={\left\|\nabla u\right\|}_{1}+\frac{\lambda }{2}{\left\|u-f\right\|}_{2}^{2}. \end{array}$$

It is composed of a regular term composed of *L*1 norm and a data term composed of quadratic norm, where *u* is the target solution, and *f* is the input image with noise. When ‖∇*u*‖_1_ in formula (31) is replaced with the form of formula (24), the original dual form of the ROF denoising model can be obtained:

(32) $$\begin{array}{c} \underset{u}{\min}\underset{p\in R^{2MN}}{\max}\left\{E\left(u,p\right)≜\langle p,\nabla u\rangle -\delta_{p}\left(p\right)+\frac{\lambda }{2}{\left\|u-f\right\|}_{2}^{2}\right\}. \end{array}$$

In the formula, *p* is a dual variable. The convex set *Р* in formula (32) is defined as

(33) $$\begin{array}{c} P=\left\{p\in R^{2MN}:{\left\|p\right\|}_{\infty }\leq 1\right\}. \end{array}$$

Among them, ‖*p*‖_*∞*_=max_*i*,*j*_|*p*_*i*,*j*_| represents the discrete maximum norm. By comparing formula (32) with formula (16), it is not difficult to see that the original dual form of the ROF denoising model is a saddle point problem.

First, for the original dual form of the ROF denoising model (29), the partial derivative of the dual variable *p* is calculated, and the presolvent formula of the dual variable is obtained:

(34) $$\begin{array}{c} \frac{\partial E\left(u,p\right)}{\partial p}=\partial_{p}\left(\langle p,\nabla u\rangle -\delta_{p}\left(p\right)+\frac{\lambda }{2}{\left\|u-f\right\|}_{2}^{2}\right)=\nabla u. \end{array}$$

Then, we find the gradient of the dual variable *u*^*n*^ by fixing the original variable *j* at the time:

(35) $$\begin{array}{c} \frac{p^{n+1}-p^{n}}{\sigma_{p}}=\nabla u^{n}. \end{array}$$

Since *F*^*∗*^(*p*)=*δ*_*p*_(*p*) is an indicator function of a convex set, when solving *p*^*n*+1^, formula (35) needs to be projected onto the unit sphere ∏_1_(*x*)=*x*/max(1, ‖*x*‖), so the final iterative update equation for dual variables is

(36) $$\begin{array}{c} p^{n+1}=\prod_{1}\left(\delta_{p}\nabla u+p^{n}\right)=\frac{\delta_{p}\nabla u+p^{n}}{\max\left(1,\left\|\delta_{p}\nabla u+p^{n}\right\|\right)}. \end{array}$$

Similarly, by calculating the partial derivative of *u* in formula (32), the presolvent formula of the original variable *u* is obtained:

(37) $$\begin{array}{c} \frac{\partial E\left(u,p\right)}{\partial u}=\partial_{u}\left(\langle p,\nabla u\rangle -\delta_{p}\left(p\right)+\frac{\lambda }{2}{\left\|u-f\right\|}_{2}^{2}\right)\\ =\nabla \cdot p+\lambda \left(u-f\right). \end{array}$$

In the formula, 〈*p*, ∇*u*〉=〈*u*, −∇·*p*〉. By fixing the obtained *p*^*n*+1^ and performing gradient descent on the original variable, the iterative update equation of the original variable *u* can be further obtained:

(38) $$\begin{array}{c} \frac{u^{n+1}-u^{n}}{\sigma_{u}}=\nabla \cdot p+\lambda \left(u-f\right)\Rightarrow u^{n+1}\\ =\frac{\sigma_{u}\nabla \cdot p^{n+1}+u^{n}+\sigma_{u}\lambda f}{1+\lambda \sigma_{u}}. \end{array}$$

Finally, when the algorithm meets the preset iteration termination condition, the global optimal solution of the original variable *u* can be obtained.

Taking the optimization problem of the ROF denoising model as an example, the update of the dual variables (formulas (31) and (32)) mainly includes the point-by-point gradient operation of the original variable and the elementwise projection operation of the variable. We assume that the solution of the energy function is two-dimensional. In the discrete case, the original variable can be expressed as *u* ∈ *R*^*M*×*N*^, where *M* and *N*, respectively, represent the size in each dimension. All the elements in *u* (*i* = *x* + My) are written in the form of a column vector, namely,

(39) $$\begin{array}{c} u={\left[u_{m,n}\right]}_{M\times N}=\left[\begin{array}{cccc} u_{1,1} & u_{1,2} & \ldots & u_{1,n}\\ u_{2,1} & u_{2,2} & \ldots & u_{2,n}\\ \ldots & \ldots & \ldots & \ldots \\ u_{m,1} & u_{m,2} & \ldots & u_{m,n} \end{array}\right]⟶{\left[u_{i}\right]}_{MN\times 1}\\ =\left[\begin{array}{c} u_{1,1}\\ u_{1,2}\\ \ldots \\ u_{1,n}\\ u_{2,1}\\ \ldots \\ u_{m,1}\\ \ldots \\ u_{m,n} \end{array}\right]. \end{array}$$

The gradient operation of the original variable can be defined as

(40) $$\begin{array}{c} \nabla u≜\left(\begin{array}{ccccccc} \frac{\partial }{\partial x} & 0 & \cdots & 0 & 0 & \cdots & 0\\ 0 & \frac{\partial }{\partial x} & \cdots & 0 & 0 & \cdots & 0\\ \cdots & \cdots & \cdots & \cdots & \cdots & \cdots & \cdots \\ 0 & \cdots & \cdots & \cdots & \cdots & \cdots & \frac{\partial }{\partial x}\\ \frac{\partial }{\partial x} & 0 & \cdots & 0 & 0 & \cdots & 0\\ \cdots & \frac{\partial }{\partial x} & \cdots & 0 & 0 & \cdots & 0\\ \cdots & \cdots & \cdots & \cdots & \cdots & \cdots & \cdots \\ 0 & \cdots & \cdots & \cdots & \cdots & \cdots & \frac{\partial }{\partial x} \end{array}\right)\left[\begin{array}{c} u_{1,1}\\ u_{1,2}\\ \cdots \\ u_{1,n}\\ u_{2,1}\\ \cdots \\ u_{m,1}\\ \cdots \\ u_{m,n} \end{array}\right]=\left(\begin{array}{c} u_{1,1}^{x}\\ u_{1,2}^{x}\\ \cdots \\ u_{1,n}^{x}\\ u_{m,1}^{x}\\ \cdots \\ u_{m,n}^{x}\\ u_{1,1}^{y}\\ u_{1,2}^{y}\\ \cdots \\ u_{1,n}^{y}\\ u_{m,1}^{y}\\ \cdots \\ u_{m,n}^{y} \end{array}\right). \end{array}$$

Moreover, the partial derivatives ∂/∂*x* and ∂/∂*y* in each dimension can be realized by forward difference.

## 4. Correction of Ballet Training Movements Based on Digital Feature Recognition Technology

**Figure 5:**
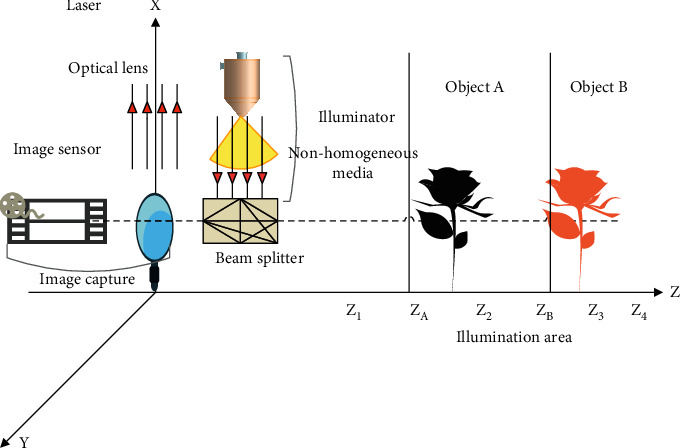
The law of digital imaging.

This article combines digital feature recognition technology to construct a ballet training movement correction system. The technical imaging principle is shown in Figure 5. When the laser transmitter does not emit a laser beam to the target, the laser speckle is formed by the scattering of the medium, and the laser speckle is projected on the target. In this process, laser speckle is a random process, and probability statistics are used to find the movement law and intensity distribution of the speckle.

**Figure 6:**
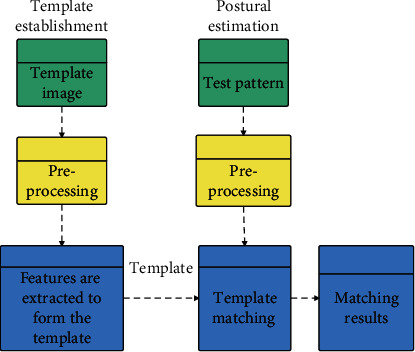
The overall process of ballet movement recognition method.

The process of the whole ballet training movement recognition method is shown in Figure 6.

**Figure 7:**
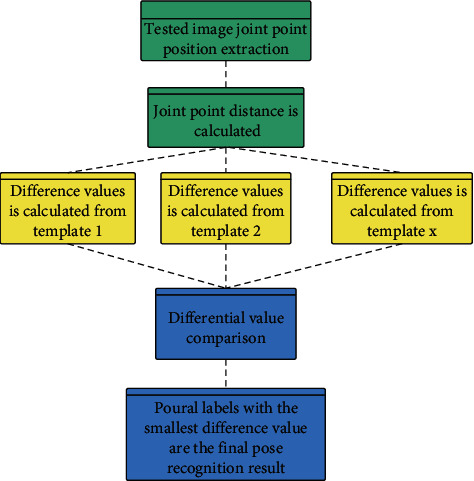
The process of ballet movement recognition system.

The correction system in this paper mainly inputs the ballet training action recognition result into the system to compare with the standard action and judge the rationality of its action. Therefore, the process of the ballet movement system proposed in this paper is shown in Figure 7 below.

**Figure 8:**
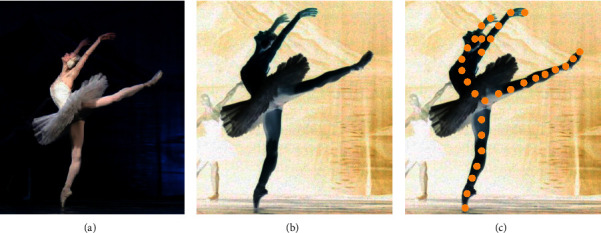
The effect diagram of ballet dance training movement correction. (a) Original ballet training image. (b) Background elimination. (c) Feature recognition.

The system built in this paper can recognize the pictures and videos of ballet training and can also perform real-time action recognition and correction for ballet training.  Figure 8 shows the effect of the system's ballet dance training action correction.

On the basis of the above analysis, the model proposed in this paper is verified. First, the digital feature recognition technology proposed in this paper is evaluated, and the statistical test results are shown in Table 1.

On this basis, the correction effect of ballet training is analyzed, and the statistical test results are shown in Table 2.

In general, the experimental research results verify that the digital feature recognition technology proposed in this paper can play an important role in ballet movement recognition and has a good motion correction effect.

## 5. Conclusion

Most of the students have not received systematic ballet dynamic technical movement training before entering the school, resulting in relatively weak students' basic skills and basic skills and relatively poor acceptance of learning new movements and new techniques. Ballet's dynamic technology has undergone constant casting and tempering by countless ancestors and constant innovations, and a very scientific and standardized training system has been formed. Moreover, it has accumulated a wealth of “clinical” experience in terms of training content and training methods. On this basis, this paper combines digital feature recognition technology to carry out ballet training movement correction analysis and combines the actual needs of actual ballet movement correction to conduct research and proposes a corresponding intelligent training correction system to improve the effect of ballet training. The experimental research results verify that the digital feature recognition technology proposed in this paper can play an important role in ballet movement recognition and has a good motion correction effect.

## Figures and Tables

**Table 1:** Digital feature recognition results of the system.

No	Feature recognition	No	Feature recognition	No	Feature recognition

1	89.7	17	89.8	33	83.1
2	84.9	18	91.0	34	88.7
3	86.2	19	92.8	35	87.5
4	84.4	20	93.2	36	87.9
5	84.5	21	90.7	37	88.1
6	92.6	22	89.5	38	90.2
7	86.4	23	88.5	39	89.5
8	90.8	24	93.8	40	83.6
9	87.8	25	90.3	41	88.4
10	85.7	26	85.4	42	90.6
11	88.3	27	93.4	43	93.5
12	85.3	28	90.1	44	89.0
13	92.7	29	88.2	45	90.7
14	88.4	30	87.2	46	92.2
15	86.8	31	84.0	47	88.5
16	83.2	32	93.8	48	88.2

**Table 2:** Corrective effect of ballet training movement of the system.

No	Action correction	No	Action correction	No	Action correction

1	89.0	17	87.2	33	84.1
2	88.7	18	90.6	34	85.7
3	86.1	19	88.8	35	88.0
4	88.9	20	84.6	36	86.7
5	83.2	21	82.6	37	83.5
6	89.4	22	81.8	38	88.9
7	89.1	23	91.5	39	82.8
8	85.5	24	84.7	40	79.4
9	85.4	25	82.1	41	87.8
10	81.9	26	79.6	42	79.9
11	81.5	27	85.5	43	80.6
12	79.8	28	91.9	44	82.4
13	87.0	29	87.3	45	91.7
14	85.4	30	81.2	46	85.4
15	90.7	31	83.4	47	79.4
16	80.7	32	91.9	48	89.0

## Data Availability

The labeled dataset used to support the findings of this study is available from the corresponding author upon request.
